# Post-discharge VTE prophylaxis after bariatric surgery: balancing bleeding risk and thrombosis prevention in 275,843 patients

**DOI:** 10.1007/s00464-026-12954-8

**Published:** 2026-06-08

**Authors:** Andrew P. Gillikin, Emily P. Rabinovich, Juan S. Barajas-Gamboa, Valentin Mocanu, Jerry T. Dang, Cullen Carter, Peter Hallowell, Bruce Schirmer, Thomas H. Shin

**Affiliations:** 1 Department of Surgery, University of Virginia School of Medicine, Charlottesville, VA USA; 2 Digestive Diseases and Surgery Institute, Cleveland Clinic, Cleveland, OH USA; 3 Department of Surgery, University of Virginia School of Medicine, 1300 Jefferson Park Ave, 4th Floor, Charlottesville, VA 22903 USA

**Keywords:** Bariatric surgery, Venous thromboembolism (VTE), Post-discharge prophylaxis, Anticoagulation, Extended thromboprophylaxis

## Abstract

**Background:**

Post-discharge venous thromboembolism (VTE) chemoprophylaxis after metabolic and bariatric surgery is variably implemented in clinical practice, although the optimal pharmacologic agent and dosing regimen remain unclear. This study characterized national practice patterns in VTE chemoprophylaxis and evaluated the associations of commonly prescribed post-discharge prophylactic regimens with VTE and bleeding outcomes.

**Methods:**

A retrospective analysis of a national database identified adult patients undergoing primary sleeve gastrectomy (*n* = 184,587) or Roux-en-Y gastric bypass (*n* = 91,256) between 2018 and 2025. The primary outcomes were VTE and bleeding events through 90 postoperative days (POD). Multivariable logistic regression and propensity score matching were used to evaluate the associations of common chemoprophylaxis regimens with VTE and bleeding.

**Results:**

Among 275,843 patients undergoing metabolic and bariatric surgery, 63,744 (23.1%) received post-discharge chemoprophylaxis. Overall post-discharge VTE incidence was 0.45% at POD30 and 0.61% at POD90. Enoxaparin 40 mg once daily was the most frequently prescribed regimen (35.8%; *n* = 22,799). On multivariable analysis, apixaban 2.5 mg twice daily (OR 0.62, 95% CI 0.41–0.90), rivaroxaban 10 mg once daily (OR 0.55, 95% CI 0.26–0.99), and enoxaparin 40 mg twice daily (OR 0.72, 95% CI 0.58–0.88) were independently associated with lower odds of VTE through POD90 without increased odds of bleeding compared with no prophylaxis. In propensity score–matched analyses, 90-day VTE incidence was lower for apixaban 2.5 mg twice daily (0.41% vs. 0.64%; *p* = 0.050; *q* = 0.050) and enoxaparin 40 mg twice daily (0.53% vs. 0.75%; *p* = 0.003) compared with matched controls.

**Conclusions:**

Post-discharge VTE chemoprophylaxis after metabolic and bariatric surgery varies substantially, with meaningful differences in safety and effectiveness across regimens. In propensity score–matched analyses, apixaban 2.5 mg twice daily and enoxaparin 40 mg twice daily were each associated with lower 90-day VTE incidence compared with matched controls, without significant increase in bleeding. Prospective randomized studies are needed to define optimal patient selection, regimen selection, and therapy duration.

**Supplementary Information:**

The online version contains supplementary material available at 10.1007/s00464-026-12954-8.

Venous thromboembolism (VTE) remains an uncommon but clinically significant complication after metabolic and bariatric surgery (MBS). Although absolute event rates have been reported in approximately 0.5% of patients within postoperative day (POD) 30, VTE contributes meaningfully to postoperative morbidity and mortality after MBS, with over 80% of events occurring after hospital discharge [1–3]. Importantly, patients who develop postoperative VTE experience a nearly 14-fold increase in 30-day mortality compared with those who do not [4]. Individual patient characteristics such as history of prior VTE, postoperative complications, increasing body mass index (BMI), older age, male sex, longer operative time, and pre-existing cardiopulmonary comorbidities have all been reported in association with increased postoperative thromboembolic risk in this patient population [5–9].

The predominance of post-discharge VTE events has made extended chemoprophylaxis a central consideration in postoperative management after MBS. However, the decision to prescribe post-discharge prophylaxis remains complex, as any potential reduction in thrombotic events must be balanced against the risk of postoperative bleeding in this patient population [10–12]. Current guidelines support an individualized approach to post-discharge VTE chemoprophylaxis after bariatric surgery, and extended prophylaxis has generally been considered for selected high-risk patients, including those with prior VTE, known hypercoagulable states, limited baseline ambulation, or other major thrombotic risk factors [4]. However, despite this broad consensus favoring individualization, there remains limited evidence to guide optimal patient selection, choice of pharmacologic agent, dosing regimen, and duration of therapy [13–16]. In particular, the comparative role of low-molecular-weight heparin (LMWH) and direct oral anticoagulants (DOACs), as well as the optimal dose intensity of pharmacologic prophylaxis, remains unclear in patients with severe obesity [17].

National prescribing patterns for post-discharge VTE chemoprophylaxis after metabolic and bariatric surgery, as well as the comparative safety and effectiveness of commonly used regimens, remain incompletely defined. Despite the widespread use of post-discharge chemoprophylaxis, there is a paucity of large-scale comparative data supporting an evidence-based prophylactic regimen. Using a large national database, the present study characterized contemporary national practice patterns and evaluated the associations of commonly prescribed post-discharge prophylactic regimens with VTE and bleeding outcomes.

## Materials and methods

### Dataset

Deidentified data were extracted from Epic Cosmos, an aggregated electronic health record (EHR) dataset collated from 1903 participating hospitals and 42,400 clinics across the United States. The available data include patient demographics, diagnoses, procedures, medications, laboratory test values, and encounters at participating institutions.

### Study design and cohort

This retrospective cohort study included adults with BMI ≥ 35 kg/m^2^ who underwent either laparoscopic sleeve gastrectomy (LSG) or Roux-en-Y gastric bypass (LRYGB) between January 1, 2018, and April 1, 2025. LSG and LRYGB were identified using Current Procedural Terminology (CPT-4) codes (Supplemental Table 1). The index date for each patient was defined as the date of the first qualifying surgery within the inclusion period. Post-discharge chemoprophylaxis was defined as any outpatient order for an anticoagulant with a start date between 14 days before and one day after hospital discharge, to account for variability in prescribing and dispensing practices. Seven chemoprophylaxis regimens accounting for approximately 90% of all anticoagulant orders were selected for inclusion in subsequent analyses. Less frequently prescribed regimens were excluded from regimen-level analyses due to limited comparative sample size. Patients without a post-discharge anticoagulation prescription served as the control group. Exclusion criteria included younger than 18 years at the index date, preoperative BMI < 35, missing age or sex data, and residence outside of the US.

We examined several covariates, including sociodemographic characteristics (age, sex, race, ethnicity, Social Vulnerability Index [SVI] score), preoperative comorbidities (atrial fibrillation, prior VTE, prior anticoagulation, history of major cardiovascular events) using International Classification of Diseases, 10th revision diagnostic codes (ICD-10), and surgical history (prior abdominal surgery, revisions during study period). The full list of covariates and related codes is provided in Supplemental Table 2.

### Study endpoints

The primary outcome was post-discharge VTE, defined as an ICD-10 diagnosis of deep venous thrombosis, pulmonary embolism, or portal venous thrombosis during an encounter occurring after the index admission with a new anticoagulant prescription (Supplemental Table 2). The incidence of VTE at postoperative days (POD) 30, 60, and 90 was assessed by chemoprophylaxis regimen. By requiring both a new ICD-10 VTE diagnosis and a concurrent new anticoagulant prescription, this composite definition was designed to enhance specificity for clinically significant post-discharge VTE events requiring therapeutic intervention, while potentially under-ascertaining events managed conservatively or occurring in patients already receiving therapeutic anticoagulation for other indications. The secondary outcome was major bleeding, defined by ICD-10 diagnoses of intracranial hemorrhage (I60–I62), gastrointestinal hemorrhage (K92), and hemorrhage not elsewhere classified (R58). Minor bleeding events (epistaxis [R04.0] and postmenopausal bleeding [N95.0]) were excluded from the primary safety analysis, as these events were infrequent, clinically distinct from major hemorrhagic complications, and not significantly associated with the prophylaxis regimen in preliminary analyses (Supplemental Table 2).

### Statistical analysis

We used descriptive statistics, including median and interquartile range (IQR), to summarize the characteristics of the study population. Independent sample *t*-tests were employed for statistical comparison between continuous variables, and *χ*^2^ tests were used to compare categorical variables. Two-sided *p*-values < 0.05 were considered statistically significant. We used multivariable logistic regression to estimate the effect of post-discharge chemoprophylaxis regimen on odds of VTE and bleeding at POD30, 60, and 90 compared to no post-discharge prophylaxis, adjusting for potential confounders (age, sex, preoperative BMI, procedure, and comorbidities) identified a priori (Supplemental Table 2). Given the number of statistical comparisons evaluated, Benjamini–Hochberg false discovery rate (FDR) correction was applied to propensity score-matched analyses. FDR-adjusted *q*-values are reported alongside unadjusted *p*-values.

To account for potential confounding by treatment indication, propensity score matching was performed for the three most common regimens using 3:1 nearest-neighbor matching with a caliper of 0.2, matching each patient on a particular post-discharge prophylaxis regimen to three controls who were not on anticoagulation following discharge. Characteristics used for the propensity score models included age, sex, preoperative BMI, surgery type, history of VTE, and prior anticoagulation. Treatment effects were estimated using generalized linear models, and cluster-robust standard errors were calculated with clustering on matched pairs to account for within-pair dependency induced by matching.

In accordance with the CMS cell size suppression policy, values derived from cells containing fewer than 11 patients were masked to prevent re‑identification such that the smallest derived count was ≥ 11 patients. For regimens with smaller sample sizes, total cohort counts were also masked to allow reporting of event incidences while maintaining compliance with suppression requirements.

The study was conducted in accordance with the Strengthening the Reporting of Observational Studies in Epidemiology (STROBE) reporting guidelines for cohort studies and an exempt determination by the University of Virginia Institutional Review Board. All analyses were conducted with the use of R software (R Foundation, version 4.4.1).

## Results

### Baseline characteristics and national trends in post-discharge VTE chemoprophylaxis

A total of 275,843 adult patients with a previous history of LSG (184,587 patients) or LRYGB (91,256 patients) were identified. Rates of post-discharge VTE during the first 30, 60, and 90 PODs were 0.45%, 0.55%, and 0.61%, respectively. Table 1 summarizes patient demographics and postoperative events according to the incidence of post-discharge VTE during the first 90 PODs. Patients who developed VTE by POD90 were more likely to be older, male, have a higher BMI, and have a previous VTE.

**Table 1:** Patient characteristics and perioperative events according to venous thromboembolism within 90 postoperative days

Characteristic	No VTE (*n* = 274,170)	VTE (*n* = 1673)	*p*
Age (mean years ± SD)	43.8 ± 11.8	47.0 ± 12.0	< 0.001
Female	226,983 (82.8)	1297 (77.5)	< 0.001
Race			< 0.001
Black/African American	70,332 (25.7)	614 (36.7)	
American Indian or Alaska Native	4063 (1.5)	22 (1.3)	
White	182,333 (66.5)	955 (57.1)	
Other/unknown	17,442 (6.3)	82 (4.9)	
RUCA code (mean ± SD)	1.88 ± 2.01	1.73 ± 1.86	< 0.001
Social vulnerability index (mean ± SD)	0.60 ± 0.28	0.61 ± 0.27	0.112
Financial coverage			< 0.001
Medicaid	32,808 (12.0)	204 (12.2)	
Medicare	18,638 (6.8)	210 (12.6)	
Miscellaneous/self-pay	222,724 (81.2)	1259 (75.2)	
Preoperative BMI (mean ± SD)	45.8 ± 7.56	47.3 ± 8.52	< 0.001
Preoperative weight (lbs., mean ± SD)	280 ± 56.6	294 ± 62.7	< 0.001
Prior VTE	9197 (3.4)	473 (28.3)	< 0.001
Prior anticoagulation	17,157 (6.3)	341 (20.4)	< 0.001
Diabetes	73,945 (27.0)	517 (30.9)	< 0.001
Atrial fibrillation	7266 (2.7)	77 (4.6)	< 0.001
Anemia	67,038 (24.5)	491 (29.3)	< 0.001
Myocardial infarction	2947 (1.1)	38 (2.3)	< 0.001
Renal failure	17,514 (6.4)	212 (12.7)	< 0.001
Dialysis	1410 (0.5)	18 (1.1)	0.003
Past abdominal surgery	26,679 (15.8)	7694 (19.4)	< 0.001
Operation			0.435
Laparoscopic sleeve gastrectomy	183,452 (66.9)	1135 (67.8)	
Laparoscopic Roux-en-Y gastric bypass	90,718 (33.1)	538 (32.2)	
Perioperative chemoprophylaxis	206,007 (75.1)	1297 (77.5)	0.026
Post-discharge chemoprophylaxis	63,235 (23.1)	509 (30.4)	< 0.001
Revisional surgery during study period	4432 (1.6)	46 (2.7)	< 0.001
Surgical site infection by post-op day 30	1410 (0.5)	41 (2.5)	< 0.001
Mortality by post-op day 30	183 (0.1)	7 (0.4)	< 0.001

Significance based on independent sample *t*-tests or *χ*^2^ test as appropriate; incidence denoted with percent unless otherwise noted

*RUCA* rural–urban commuting area, *BMI* body mass index, *VTE* venous thromboembolism, *SD* standard deviation

**Fig. 1:**
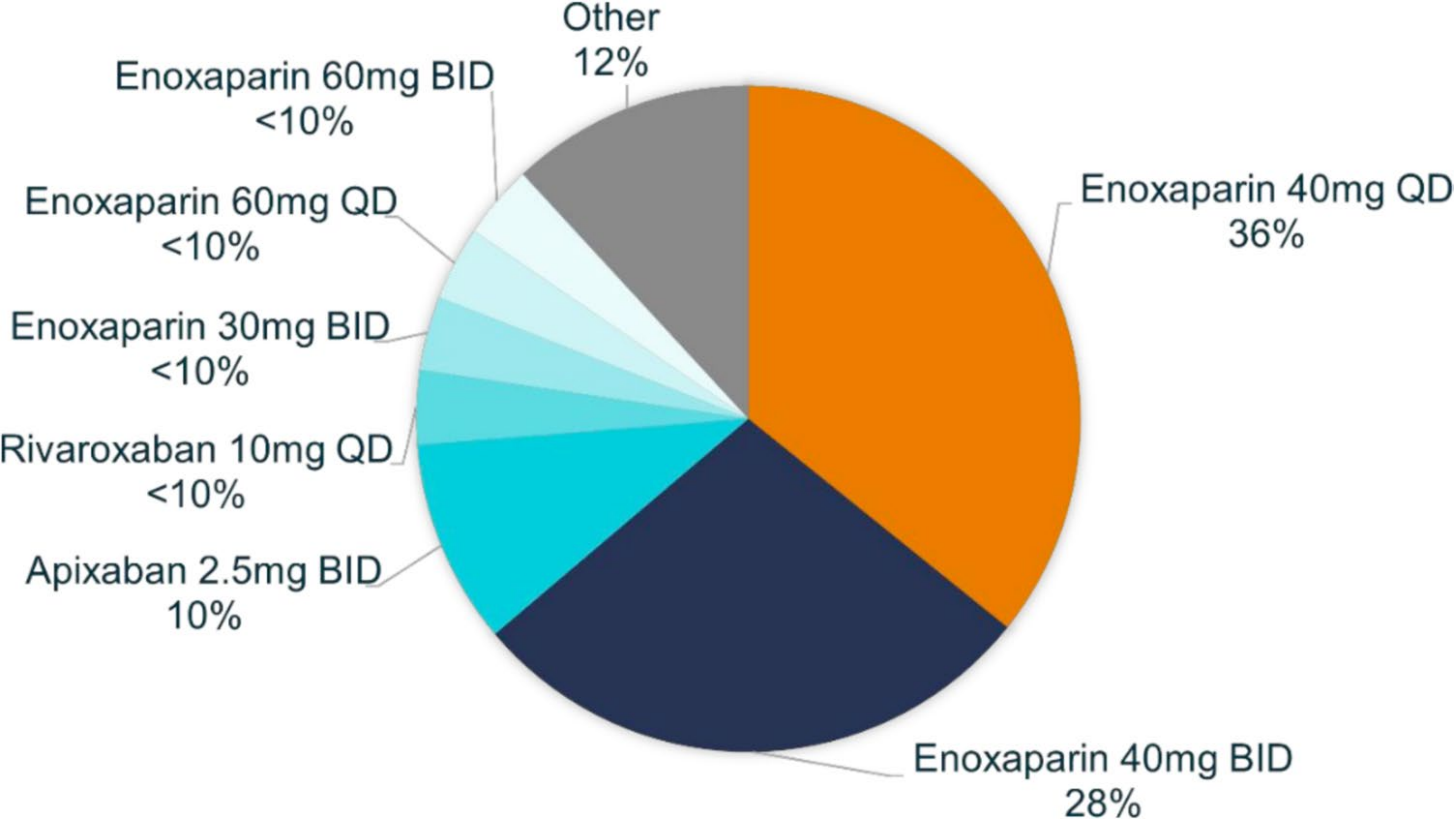
Distribution of post-discharge anticoagulation regimens

Overall, 63,744 patients (23.1%) were prescribed post-discharge chemoprophylaxis. The seven most common regimens included enoxaparin 40 mg daily (35.8%; *n* = 22,799), enoxaparin 40 mg twice daily (28.0%; *n* = 17,879), apixaban 2.5 mg twice daily (9.9%; *n* = 6332), enoxaparin 60 mg twice daily (< 10%), rivaroxaban 10 mg daily (< 10%), enoxaparin 30 mg twice daily (< 10%), and enoxaparin 60 mg daily (< 10%; Fig. 1). Table 2 compares demographic and perioperative data for patients who received any of these seven regimens of interest (*n* = 56,171) versus those who received no post-discharge chemoprophylaxis (*n* = 212,099). An additional 7573 patients who received less commonly prescribed regimens outside of these seven were excluded from regimen-level analyses. Patients prescribed one of these post-discharge chemoprophylactic regimens shared similar characteristics to those who developed VTE by POD90, including older age, male sex, higher BMI, and a greater likelihood of prior VTE. The proportion of patients who developed post-discharge VTE by POD90 was similar between those who received one of the regimens of interest and those who received no post-discharge chemoprophylaxis (0.55% vs. 0.55%, *p* = 0.996).

**Table 2:** Patient characteristics and perioperative events according to post-discharge chemoprophylaxis status

Characteristic	No Rx (*n* = 212,099)	Rx (*n* = 56,171)	*p*
Age (mean years ± SD)	43.7 ± 11.7	44.0 ± 12.0	< 0.001
Female	177,320 (83.6)	45,286 (80.6)	< 0.001
Race			< 0.001
Black/African American	52,865 (24.9)	15,897 (28.3)	
American Indian or Alaska Native	3067 (1.5)	916 (1.6)	
White	143,024 (67.4)	35,540 (63.3)	
Other/unknown	13,143 (6.2)	3818 (6.8)	
RUCA code (mean ± SD)	1.92 ± 2.04	1.75 ± 1.90	< 0.001
Social vulnerability index (mean ± SD)	0.60 ± 0.28	0.60 ± 0.28	0.675
Financial coverage			< 0.001
Medicaid	25,119 (11.8)	7014 (12.5)	
Medicare	14,057 (6.6)	3815 (6.8)	
Miscellaneous/self-pay	172,923 (81.6)	45,342 (80.7)	
Preoperative BMI (mean ± SD)	45.1 ± 7.01	47.9 ± 8.80	< 0.001
Preoperative weight (lbs., mean ± SD)	276 ± 53.0	295 ± 64.8	< 0.001
Prior VTE	5038 (2.4)	3340 (5.9)	< 0.001
Prior anticoagulation	11,650 (5.5)	4136 (7.4)	< 0.001
Diabetes	56,215 (26.5)	15,625 (27.8)	< 0.001
Atrial fibrillation	5169 (2.4)	1242 (2.2)	0.002
Anemia	50,680 (23.9)	14,574 (25.9)	< 0.001
Myocardial infarction	2191 (1.0)	603 (1.1)	0.414
Renal failure	12,722 (6.0)	3908 (7.0)	< 0.001
Dialysis	1095 (0.5)	190 (0.3)	< 0.001
Past abdominal surgery	38,675 (18.2)	11,531 (20.5)	< 0.001
Operation			0.016
Laparoscopic sleeve gastrectomy	141,442 (66.7)	37,761 (67.2)	
Laparoscopic Roux-en-Y gastric bypass	70,657 (33.3)	18,410 (32.8)	
Perioperative chemoprophylaxis	156,949 (74.0)	44,329 (78.9)	0.026
Revisional surgery during study period	3491 (1.6)	852 (1.5)	0.033
VTE by post-op day 90	1164 (0.5)	309 (0.5)	0.996
Surgical site infection by post-op day 30	1070 (0.5)	324 (0.6)	0.037
Mortality by post-op day 30	146 (0.1)	32 (0.1)	0.379

Significance based on independent sample *t*-tests or *χ*^2^ test as appropriate; incidence denoted with percent unless otherwise noted

*Rx* indicates patients who received any of the seven post-discharge chemoprophylaxis regimens of interest, *No Rx* indicates no post-discharge chemoprophylaxis, *RUCA* rural–urban commuting area, *BMI* body mass index, *VTE* venous thromboembolism, *SD* standard deviation

### Post-discharge VTE and bleeding by regimen

There were no significant differences in postoperative VTE between surgery types: by POD90, VTE occurred in 0.61% of patients after LSG (*n* = 1135) and 0.60% after LRYGB (*n* = 538; *p* = 0.435; Table 1). Bleeding rates were low overall but varied by procedure and prophylaxis exposure. The incidence of VTE at POD30, 60, and 90 by post-discharge chemoprophylaxis regimen is provided in Table 3 to provide additional context for regimen-specific event rates in the cohort containing the seven most commonly prescribed regimens. Table 3 also provides the incidence of bleeding events by post-discharge chemoprophylaxis regimen. Apixaban 2.5 mg twice daily, rivaroxaban 10 mg daily, and enoxaparin 60 mg daily were not associated with a significant increase in bleeding incidence at any postoperative interval compared with no post-discharge chemoprophylaxis. Enoxaparin 40 mg twice daily was associated with higher major bleeding incidence at POD30 (0.36% vs. 0.28%; *p* = 0.041), POD60 (0.45% vs. 0.34%; *p* = 0.030), and POD90 (0.52% vs. 0.40%; *p* = 0.019). Enoxaparin 30 mg twice daily was associated with increased major bleeding at POD30 (0.56% vs. 0.28%; *p* = 0.030), with no significant difference at later intervals. Enoxaparin 60 mg twice daily was associated with substantially higher major bleeding incidence at all postoperative intervals through POD90 (1.06% vs. 0.40%; *p* < 0.001). In LSG, major bleeding occurred in 0.22% without post-discharge chemoprophylaxis compared to 0.26% with prophylaxis (*p* = NS; RR 1.18). In LRYGB, major bleeding was higher overall and significantly more frequent with prophylaxis at all intervals through POD90 (POD30:0.70% vs. 0.53%, *p* = 0.011; POD60:0.85% vs. 0.65%, *p* = 0.005; POD90:0.99% vs. 0.76%, *p* = 0.003; Supplemental Table 3).

**Table 3:** Incidence of venous thromboembolism and bleeding events within 30, 60, and 90 postoperative days (POD) according to post-discharge chemoprophylaxis regimen

Regimen	Venous thromboembolism						Major bleeding event					
	POD 30		POD 60		POD 90		POD 30		POD 60		POD 90	
	*n* (%)	*p*	*n* (%)	*p*	*n* (%)	*p*	*n* (%)	*p*	*n* (%)	*p*	*n* (%)	*p*
None (reference)	898 (0.42)	–	1079 (0.51)	–	1164 (0.55)	–	585 (0.28)	–	730 (0.34)	–	850 (0.40)	–
Apixaban 2.5 mg BID	14 (0.22)	0.018	22 (0.35)	NS	26 (0.41)	NS	13 (0.21)	NS	14 (0.22)	NS	16 (0.25)	NS
Rivaroxaban 10 mg QD	*** (< 0.18)	0.014	*** (0.21)	0.037	*** (0.32)	NS	*** (0.18)	NS	*** (0.21)	NS	*** (0.28)	NS
Enoxaparin 40 mg QD	84 (0.37)	NS	112 (0.49)	NS	123 (0.54)	NS	71 (0.31)	NS	89 (0.39)	NS	111 (0.49)	NS
Enoxaparin 40 mg BID	58 (0.32)	NS	81 (0.45)	NS	95 (0.53)	NS	65 (0.36)	0.041	80 (0.45)	0.030	93 (0.52)	0.019
Enoxaparin 30 mg BID	*** (0.36)	NS	*** (0.51)	NS	*** (0.51)	NS	*** (0.56)	0.030	*** (0.61)	NS	*** (0.71)	NS
Enoxaparin 60 mg QD	*** (0.96)	0.011	*** (1.22)	0.002	*** (1.48)	< 0.001	*** (0.26)	NS	*** (0.35)	NS	*** (0.35)	NS
Enoxaparin 60 mg BID	*** (0.50)	NS	*** (0.68)	NS	*** (0.90)	0.011	*** (0.87)	< 0.001	*** (0.90)	< 0.001	*** (1.06)	< 0.001

Significance based on *χ*^2^ test with no post-discharge chemoprophylaxis serving as comparison

*QD* once daily, *BID* twice daily

***Indicates suppressed count per CMS policy

On multivariable analysis, several post‑discharge chemoprophylaxis regimens independently predicted lower odds of VTE compared with no prophylaxis without increased bleeding risk (Table 4). Additional independent predictors of VTE at PODs 30, 60, and 90 are presented in Supplemental Table 4. Apixaban 2.5 mg twice daily was associated with significantly reduced odds of VTE through POD90 (OR 0.62 [0.41–0.90]), as were rivaroxaban 10 mg daily (OR 0.55 [0.26–0.99]) and enoxaparin 40 mg twice daily (OR 0.72 [0.58–0.88]). In contrast, enoxaparin 60 mg once daily was associated with increased odds of VTE by POD90 (OR 1.91 [1.12–3.03]), a finding likely attributable to residual confounding by indication. No significant difference in VTE odds was observed for enoxaparin 30 mg or 60 mg twice daily following multivariable adjustment. Enoxaparin 60 mg twice daily was independently associated with increased odds of bleeding at all postoperative intervals through POD90 (Table 4; OR 2.11 [1.46–2.94]). Notably, despite the higher unadjusted major bleeding incidence observed with enoxaparin 40 mg twice daily, this association was not significant after multivariable adjustment at any interval (OR 1.13 [0.86–1.45] at POD30; 1.12 [0.88–1.40] at POD60; 1.13 [0.91–1.40] at POD90), suggesting that the unadjusted signal may reflect preferential prescribing of twice-daily dosing to patients with higher baseline risk rather than a direct causal effect. No post‑discharge DOAC regimen was associated with increased adjusted odds of major bleeding, and no significant association with major bleeding was observed for enoxaparin 30 mg twice daily, enoxaparin 40 mg once daily, or enoxaparin 60 mg once daily following adjustment (Table 4). Additional independent predictors of bleeding events at POD30, 60, and 90 are presented in Supplemental Table 5.

**Table 4:** Adjusted odds of venous thromboembolism and bleeding events within 30, 60, and 90 postoperative days (POD) according to post-discharge chemoprophylaxis regimen

Regimen	Venous thromboembolism			Major bleeding event		
	POD 30	POD 60	POD 90	POD 30	POD 60	POD 90
	OR (95% CI)	OR (95% CI)	OR (95% CI)	OR (95% CI)	OR (95% CI)	OR (95% CI)
Apixaban 2.5 mg BID	0.44 (0.25–0.72)	0.57 (0.36–0.85)	0.62 (0.41–0.90)	0.88 (0.48–1.47)	0.76 (0.42–1.23)	0.74 (0.43–1.18)
Rivaroxaban 10 mg QD	0.23 (0.06–0.60)	0.39 (0.15–0.79)	0.55 (0.26–0.99)	1.02 (0.36–2.21)	0.97 (0.38–1.98)	1.11 (0.51–2.09)
Enoxaparin 40 mg QD	0.76 (0.60–0.94)	0.83 (0.68–1.01)	0.85 (0.70–1.02)	1.03 (0.80–1.32)	1.04 (0.83–1.29)	1.12 (0.91–1.36)
Enoxaparin 40 mg BID	0.59 (0.44–0.76)	0.66 (0.52–0.83)	0.72 (0.58–0.88)	1.13 (0.86–1.45)	1.12 (0.88–1.40)	1.13 (0.91–1.40)
Enoxaparin 30 mg BID	0.66 (0.28–1.29)	0.77 (0.38–1.36)	0.71 (0.35–1.25)	1.83 (0.94–3.18)	1.61 (0.86–2.73)	1.62 (0.91–2.66)
Enoxaparin 60 mg QD	1.64 (0.84–2.85)	1.69 (0.94–2.79)	1.91 (1.12–3.03)	0.78 (0.19–2.04)	0.84 (0.26–1.98)	0.74 (0.23–1.74)
Enoxaparin 60 mg BID	0.80 (0.46–1.27)	0.87 (0.55–1.30)	1.05 (0.70–1.50)	2.43 (1.61–3.51)	2.04 (1.36–2.92)	2.11 (1.46–2.94)

*OR* odds ratio with a given post-hospital discharge chemoprophylaxis regimen versus without any chemoprophylaxis as control arm, *CI* 95% confidence interval

To evaluate whether the altered gastrointestinal anatomy following RYGB affects DOAC-based prophylaxis outcomes, a subgroup analysis compared VTE and major bleeding events among patients prescribed apixaban 2.5 mg twice daily or rivaroxaban 10 mg daily, stratified by procedure type (Supplemental Table 6). No statistically significant differences in VTE or major bleeding incidence were observed between LSG and LRYGB at any postoperative interval for either DOAC regimen, though statistical power was limited by low absolute event rates and CMS cell suppression requirements after stratification by procedure.

### Propensity score-matched analyses

To address confounding by indication in comparisons of post-discharge prophylaxis strategies, we performed propensity score matching for the three most commonly prescribed regimens (enoxaparin 40 mg once daily, enoxaparin 40 mg twice daily, and apixaban 2.5 mg twice daily), matching each treated patient to three controls who did not receive post-discharge anticoagulation (Table 5). Covariate balance was confirmed with insignificant differences between the two groups for each model (Supplemental Fig. 1). In these matched analyses, apixaban 2.5 mg twice daily was associated with lower VTE incidence through POD30 (0.22% vs. 0.47%; *p* = 0.006, *q* = 0.027) and POD60 (0.35% vs. 0.58%; *p* = 0.018, *q* = 0.046), with a trend toward lower incidence at POD90 that did not meet statistical significance after FDR correction (0.41% vs. 0.64%; *p* = 0.050, *q* = 0.050). In contrast, enoxaparin 40 mg once daily was associated with lower VTE incidence only through POD30. None of the three studied regimens demonstrated a significant increase in bleeding odds relative to their matched control group (Table 5).

**Table 5:** Outcomes within 30, 60, and 90 postoperative days (POD) by post-discharge chemoprophylaxis regimen in propensity-matched cohorts

Regimen	Venous thromboembolism						Major bleeding event					
	POD 30		POD 60		POD 90		POD 30		POD 60		POD 90	
	*n* (%)	OR (95%CI)	*n* (%)	OR (95%CI)	*n* (%)	OR (95%CI)	*n* (%)	OR (95%CI)	*n* (%)	OR (95%CI)	*n* (%)	OR (95%CI)
None	90 (0.47)	–	111 (0.58)	–	121 (0.64)	–	46 (0.24)	–	52 (0.27)	–	58 (0.31)	–
Apixaban 2.5 mg BID	14 (0.22)	0.47 (0.27–0.82)	22 (0.35)	0.59 (0.38–0.94)	26 (0.41)	0.64 (0.42–0.98)	13 (0.21)	0.85 (0.46–1.57)	14 (0.22)	0.81 (0.45–1.46)	16 (0.25)	0.83 (0.48–1.44)
None	330 (0.48)	–	409 (0.60)	–	446 (0.65)	–	206 (0.30)	–	261 (0.38)	–	302 (0.44)	–
Enoxaparin 40 mg QD	84 (0.37)	0.76 (0.60–0.97)	112 (0.49)	0.82 (0.67–1.01)	123 (0.54)	0.83 (0.68–1.01)	71 (0.31)	1.03 (0.79–1.35)	89 (0.39)	1.02 (0.80–1.30)	111 (0.49)	1.10 (0.89–1.37)
None	288 (0.54)	–	361 (0.67)	–	401 (0.75)	–	169 (0.32)	–	215 (0.40)	–	252 (0.47)	–
Enoxaparin 40 mg BID	58 (0.32)	0.60 (0.45–0.80)	81 (0.45)	0.67 (0.53–0.86)	95 (0.53)	0.71 (0.57–0.89)	65 (0.36)	1.15 (0.87–1.54)	80 (0.45)	1.12 (0.86–1.44)	93 (0.52)	1.11 (0.87–1.41)

*OR* odds ratio estimated by generalized linear model, *CI* 95% confidence interval

## Discussion

This study represents the first large-scale database analysis describing national practice patterns for post-discharge VTE chemoprophylaxis following metabolic and bariatric surgery. Nationwide, there is substantial variation in VTE chemoprophylaxis prescribing patterns, possibly reflective of the fact that current ASMBS guidelines recommend an individualized approach to VTE prevention. More importantly, post-discharge prophylaxis was not uniformly associated with lower odds of VTE, underscoring the importance of appropriate regimen selection. Given the low overall absolute incidence of post-discharge VTE, these findings also suggest that a more selective, risk-based approach to extended prophylaxis may be more appropriate. Notably, the present analysis was unable to account for the duration of and compliance to prescribed prophylaxis, which may contribute to the observed heterogeneity in outcomes across regimens and should be considered when interpreting these findings.

Notably, the overall incidence of post-discharge VTE by POD90 was similar between patients who received post-discharge chemoprophylaxis and those who did not (0.55% vs. 0.55%; *p* = 0.996). However, this finding should not be interpreted as evidence that prophylaxis is ineffective. As demonstrated in Table 2, patients prescribed post-discharge chemoprophylaxis had significantly higher baseline thrombotic risk, including higher preoperative BMI, greater prevalence of prior VTE (5.9% vs. 2.4%), and more frequent prior anticoagulation use (7.4% vs. 5.5%). Therefore, the observation that crude VTE rates were equivalent between treated and untreated groups, despite meaningful differences in baseline risk, is consistent with a risk-attenuating effect of prophylaxis in the treated cohort. This interpretation is further supported by the propensity score-matched analyses, in which select regimens were associated with significantly lower VTE incidence compared with risk-matched controls.

A central challenge in post-discharge VTE chemoprophylaxis is balancing thrombotic risk against bleeding risk. These findings suggest that bleeding risk may be mitigated through appropriate regimen selection, as outcomes varied meaningfully by regimen agent and dosage. Although enoxaparin 40 mg daily was the most frequently prescribed post-discharge regimen, higher-intensity low-molecular-weight heparin (LMWH) regimens did not appear to confer additional benefit. While enoxaparin 40 mg twice daily was associated with higher unadjusted major bleeding incidence at all three postoperative intervals (Table 3), this association was not significant after multivariable adjustment (Table 4), suggesting that the unadjusted signal may reflect preferential prescribing of twice-daily dosing to patients with higher baseline bleeding risk factors rather than a direct causal effect of the regimen itself. Escalation to higher enoxaparin doses did not appear to provide additional benefit or improve effectiveness. Enoxaparin 60 mg twice daily was associated with a significantly increased risk of postoperative bleeding without a significant corresponding reduction in VTE. Notably, enoxaparin 60 mg once daily was paradoxically associated with increased odds of VTE (OR 1.91 [1.12–3.03]). This finding is likely driven primarily by confounding by indication, as patients prescribed this regimen may represent a particularly high-risk subgroup, including those with prior VTE, known hypercoagulable states, or perceived inadequacy of lower-dose strategies, for whom the underlying thrombotic risk may not be fully captured by the covariates available for adjustment. Additionally, once-daily dosing of enoxaparin may not provide adequate trough anti-Xa levels in patients with severe obesity, given the accelerated clearance and increased volume of distribution of LMWH reported in this population [18, 19]. However, the relatively small size of this subgroup limits statistical precision and widens the confidence interval around the point estimate. The combination of a high-risk patient population, potentially subtherapeutic pharmacokinetics, and low proportion of patients on enoxaparin 60 mg once daily may account for this counterintuitive finding. Importantly, this observation should not be interpreted as evidence that enoxaparin 60 mg once daily causes harm, but rather that this regimen, as prescribed in real-world practice, may be disproportionately selected for patients whose baseline risk exceeds the protective capacity of a once-daily dosing interval. Accordingly, this finding should not inform clinical decision-making regarding the efficacy of enoxaparin 60 mg once daily, and should instead be interpreted as a reflection of the inherent limitations of observational treatment comparisons in the setting of risk-stratified prescribing.

Importantly, the safety profile of post-discharge chemoprophylaxis differed by procedure type. On multivariable analysis, LSG was independently associated with substantially lower odds of major bleeding compared with LRYGB (OR 0.27 [0.25–0.32]; Supplemental Table 5), representing an approximately 3.7-fold higher risk of major hemorrhagic events following gastric bypass. This differential risk may reflect the altered gastrointestinal anatomy following bypass, which exposes vulnerable anastomotic and staple lines to intraluminal anticoagulant effects. These findings suggest that prophylaxis selection may benefit from procedure-specific risk stratification, with particular attention to regimen choice and dosing in RYGB patients. Whether DOAC-based strategies, which demonstrated favorable safety profiles in the overall cohort, offer a differential advantage in RYGB patients warrants further investigation, though the present procedure-stratified DOAC analysis was limited by small event counts.

Direct oral anticoagulants (DOAC)-based regimens demonstrated favorable associations in this analysis. In propensity score–matched comparisons, apixaban 2.5 mg twice daily was associated with lower 90-day VTE incidence compared with risk-matched controls, without increased odds of bleeding. Apixaban 2.5 mg twice daily was associated with among the lowest unadjusted VTE rates and significantly reduced adjusted odds of VTE through POD90 (OR 0.62 [0.41–0.90]). Rivaroxaban 10 mg daily demonstrated a similar direction of effect (OR 0.55 [0.26–0.99]), although the confidence interval approaching unity and the relatively small sample of rivaroxaban-treated patients (< 10%) suggest that this association should be interpreted with caution pending larger confirmatory studies. Neither regimen was associated with significantly increased bleeding risk compared with no prophylaxis. A potential concern with DOAC-based prophylaxis following RYGB is altered drug absorption due to bypass of the proximal small bowel, the primary absorption site for apixaban and rivaroxaban. In the present procedure-stratified subgroup analysis, no significant differences in VTE or major bleeding outcomes were observed between LSG and LRYGB patients prescribed DOAC-based prophylaxis, although the small number of events within each cohort limits definitive conclusions. These findings do not substitute for formal pharmacokinetic evaluation, and clinicians should remain attentive to the theoretical risk of reduced DOAC bioavailability following gastric bypass. Altogether, these findings suggest that transitioning toward DOAC-based strategies, as well as optimized twice-daily enoxaparin 40 mg dosing, may warrant further prospective evaluation as post-discharge prophylactic approaches for high-risk bariatric surgery patients. Interpretation of these findings must account for the likelihood of treatment selection bias, as differences between unadjusted and adjusted outcomes across prophylactic regimens likely reflect, at least in part, preferential prescribing of post-discharge chemoprophylaxis to patients perceived to be at higher baseline risk for VTE.

The potential advantages of DOAC-based post-discharge prophylaxis extend beyond the efficacy and safety signals observed in this analysis. From a practical standpoint, oral administration eliminates the burden of daily subcutaneous self-injection, which has been identified as a barrier to medication adherence in the bariatric surgery population [20–22]. While the present analysis cannot directly assess differential adherence between oral and injectable regimens, the theoretical advantage of simplified oral dosing warrants consideration as a potential contributor to the observed real-world effectiveness of DOAC-based strategies. Furthermore, DOACs offer fixed-dose regimens that do not require anti-Xa monitoring, simplifying outpatient management. Apixaban 2.5 mg twice daily was associated with significantly reduced adjusted odds of VTE at all postoperative intervals through POD90 on multivariable analysis (OR 0.62 [0.41–0.90]). In propensity score–matched analyses, this association was confirmed at POD30 (*q* = 0.027) and POD60 (*q* = 0.046) after Benjamini–Hochberg correction for multiple comparisons. The matched comparison at POD90, while directionally consistent, did not achieve statistical significance after correction (*q* = 0.050), and should be interpreted with appropriate caution. Taken together, the convergence of the multivariable and matched analyses supports apixaban 2.5 mg twice daily as a promising candidate for post-discharge prophylaxis, though prospective validation remains warranted. As post-discharge VTE prophylaxis is predominantly managed in the ambulatory setting, these practical considerations may meaningfully influence real-world effectiveness beyond what is captured in administrative claims data.

The low absolute incidence of post-discharge VTE has important implications for the number needed to treat (NNT) and the overall burden of prophylaxis. Based on the propensity-matched analyses, the estimated NNT to prevent one VTE event at POD30 was 396 for apixaban 2.5 mg twice daily and 471 for enoxaparin 40 mg twice daily; at POD90, the corresponding NNTs were 442 and 463, respectively. These figures underscore that most patients treated with post-discharge prophylaxis will not experience a VTE event regardless of treatment, and that the decision to prescribe should weigh the potential cost, inconvenience, and bleeding risk faced by the many patients who would not have developed VTE. This consideration further supports a selective, risk-stratified approach to extended prophylaxis rather than routine universal prescribing.

### Limitations

Several limitations warrant consideration in interpreting these findings. Epic Cosmos does not capture the prescribed duration and compliance with post-discharge chemoprophylaxis. As a result, we were unable to evaluate the effect of prophylaxis duration on VTE or bleeding outcomes, or to distinguish between short-course (e.g., 7–14 days) and extended (e.g., 30+ days) prophylaxis within a given regimen. This represents a critical gap, as duration likely modifies both the efficacy and safety of any prophylactic strategy. Future studies utilizing datasets with prescription-level duration data or prospective trial designs are needed to address this question. As a retrospective EHR-based observational analysis, this study is susceptible to confounding by indication. Treatment was non-randomized and patients were likely prescribed higher-intensity or DOAC-based regimens based on perceived thrombotic risk, resulting in selection bias reflected in discrepancies between unadjusted and adjusted results. As a result, the reference group of patients not on post-discharge chemoprophylaxis may inadvertently represent a lower-risk population and comparisons against this group should not necessarily be interpreted as evidence of efficacy relative to a risk-matched untreated cohort. To address this, the study employed propensity score matching based on covariates canonically related to perioperative VTE risk which yielded results that were directionally consistent with the multivariable analysis. Epic Cosmos does not capture prescription-level details surrounding medication adherence, prophylaxis duration, compliance with weight-based regimen selection, and clinical rationale behind regimen selection. Other clinically relevant qualitative data are also unavailable, such as perioperative mobility, functional status, and nuances in recovery pathways that affect VTE prophylaxis. VTE outcomes were defined by an ICD-10 diagnosis with concomitant new anticoagulant prescription. While this enhances specificity for clinically actionable VTEs, it may underestimate other thromboembolic events that are managed conservatively or that occur in patients already receiving therapeutic anticoagulation. Additionally, the reliance on ICD-10 codes for outcomes depends on the accuracy of diagnostic coding at participating institutions. While bleeding outcomes focus on major events, the dataset does not permit further severity stratification by transfusion requirement, hospital readmission, or procedural reintervention.

## Conclusion

In the setting of metabolic and bariatric surgery, post-discharge VTE chemoprophylaxis remains highly variable in contemporary practice, with meaningful differences in safety and effectiveness across regimens. In this large national cohort, higher-intensity low-molecular-weight heparin (LMWH) regimens, particularly enoxaparin 60 mg twice daily, were associated with increased postoperative bleeding without evidence of additional protection against VTE. In contrast, DOAC-based regimens and enoxaparin 40 mg twice daily dosing were associated with more favorable safety-effectiveness profiles and may represent preferable strategies for selected high-risk patients. Prospective randomized studies are needed to further define optimal patient selection, choice of prophylactic regimen, including agent and dosing, as well as duration of therapy in this population.

## Supplementary Information

Below is the link to the electronic supplementary material.

Supplementary file1 (DOCX 353 KB)
