# Supplementary material for: Post-discharge VTE prophylaxis after bariatric surgery: balancing bleeding risk and thrombosis prevention in 275,843 patients
Source: Surg Endosc. 2026 Jun 8;40(7):6211–21. doi: 10.1007/s00464-026-12954-8 (PMC13369671; doi:10.1007/s00464-026-12954-8)
Supplement: Supplementary file 1 — Supplementary file1 (DOCX 353 KB) [file 464_2026_12954_MOESM1_ESM.docx]

**Supplemental Table 1.** Current Procedural Terminology 4^th^ edition (CPT-4) codes used

| **Procedure** | **CPT-4** |
| --- | --- |
| Laparoscopic sleeve gastrectomy | 43775 |
| Laparoscopic Roux-en-Y gastrectomy | 43644, 43645 |

**Supplemental Table 2.** Patient characteristics used as covariates, venous thromboembolism and bleeding outcomes with ICD-10 and CPT-4 codes

| **Covariates** | **Codes** |
| --- | --- |
| Diabetes mellitus | ICD-10: E08, E09, E10, E11, E13 |
| Atrial fibrillation | ICD-10: I48* |
| Anemia | Diagnosis names containing “anemia” |
| Myocardial infarction | ICD-10: I21, I22, I23 |
| Renal failure | ICD-10: I12, I13, N17, N18, N19, Z49, Z99.2  Diagnosis names containing “kidney disease” |
| Dialysis | ICD-10: Z49*, Z99.2*  Diagnosis names containing “renal dialysis” |
| Previous abdominal surgery | CPT-4: 435*, 436*, 437*, 438*, 439*, 44*, 45*, 47*, 48*, 49* |
| Bariatric surgery revision/reoperation | CPT-4: 43644, 43645, 43775, 43845, 43846, 43847, 43848 |
|  |  |
| **Outcomes** |  |
| Venous thromboembolism | ICD-10: I26, I80.1, I80.2, I80.3, I81, I82.4, I82.6, K75.1 |
| Major bleeding events |  |
| Intracranial hemorrhage | ICD-10: I60*, I61*, I62* |
| Gastrointestinal bleed | ICD-10: K92 |
| Hemorrhage, not elsewhere classified | ICD-10: R58 |
|  |  |
| Minor bleeding events |  |
| Postmenopausal bleed | ICD-10: N95.0 |
| Nosebleed | ICD-10: R04.0 |

ICD-10: International Classification of Diseases, 10^th^ revision
CPT-4: Current Procedural Terminology, 4^th^ edition

| **Bleeding Incidence, n (%)** | **LSG** | | | |  | **LRYGB** | | | |  |
| --- | --- | --- | --- | --- | --- | --- | --- | --- | --- | --- |
|  | **No Rx  (N=141442)** | **Rx  (N=37761)** | **p** | **RR** |  | **No Rx  (N=70657)** | **Rx  (N=18410)** | **p** | **RR** |  |
| Within POD30 | 208 (0.15) | 68 (0.18) | NS | 1.22 |  | 377 (0.53) | 128 (0.70) | 0.011 | 1.30 |  |
| Within POD60 | 269 (0.19) | 78 (0.21) | NS | 1.09 |  | 461 (0.65) | 156 (0.85) | 0.005 | 1.30 |  |
| Within POD90 | 311 (0.22) | 98 (0.26) | NS | 1.18 |  | 539 (0.76) | 182 (0.99) | 0.003 | 1.30 |  |

**Supplemental Table 3.** Major bleeding events within 30, 60, and 90 postoperative days (POD) by procedure and post-discharge chemoprophylaxis status

Significance based on χ² test comparing No Rx vs. Rx within each timepoint. Rx: Indicates patients who received any of the seven post-discharge chemoprophylaxis regimens of interest; No Rx: Indicates no post-discharge chemoprophylaxis; LSG: laparoscopic sleeve gastrectomy; LRYGB: laparoscopic Roux-en-Y gastric bypass; RR: relative risk

**Supplemental Table 4.** Multivariable analyses of other predictors of venous thromboembolism
at 30, 60, and 90 postoperative days (POD)

|  | **POD 30** | |  | **POD 60** | |  | **POD 90** | |  |
| --- | --- | --- | --- | --- | --- | --- | --- | --- | --- |
| **Parameter** | **OR** | **95% CI** |  | **OR** | **95% CI** |  | **OR** | **95% CI** |  |
| Age ≥ 60 | 1.24 | 1.03-1.49 |  | 1.21 | 1.02-1.41 |  | 1.27 | 1.09-1.47 |  |
| Male sex | 1.26 | 1.08-1.46 |  | 1.24 | 1.08-1.41 |  | 1.24 | 1.09-1.41 |  |
| LSG (vs. LRYGB) | 1.14 | 1.00-1.31 |  | 1.12 | 0.99-1.26 |  | 1.11 | 0.99-1.24 |  |
| SVI | 1.28 | 1.02-1.59 |  | 1.18 | 0.97-1.44 |  | 1.18 | 0.98-1.43 |  |
| Pre-op BMI ≥ 40 | 1.22 | 1.05-1.42 |  | 1.26 | 1.09-1.45 |  | 1.29 | 1.13-1.48 |  |
| Peri-op prophylaxis | 1.11 | 0.97-1.28 |  | 1.08 | 0.95-1.23 |  | 1.07 | 0.95-1.21 |  |
| Prior VTE | 7.49 | 6.31-8.86 |  | 7.99 | 6.88-9.25 |  | 7.99 | 6.94-9.18 |  |
| Prior anticoagulation | 1.24 | 1.01-1.51 |  | 1.39 | 1.17-1.65 |  | 1.48 | 1.25-1.73 |  |
| History of diabetes | 0.84 | 0.73-0.97 |  | 0.94 | 0.82-1.06 |  | 0.96 | 0.85-1.08 |  |
| History of AFib | 0.92 | 0.65-1.27 |  | 0.85 | 0.62-1.13 |  | 0.85 | 0.63-1.11 |  |
| History of anemia | 0.97 | 0.84-1.11 |  | 0.96 | 0.84-1.09 |  | 0.99 | 0.88-1.12 |  |
| History of MI | 1.39 | 0.89-2.08 |  | 1.22 | 0.81-1.76 |  | 1.18 | 0.80-1.67 |  |
| History of renal failure | 1.01 | 0.79-1.28 |  | 1.28 | 1.05-1.55 |  | 1.33 | 1.11-1.59 |  |
| History of dialysis | 0.91 | 0.40-1.78 |  | 0.93 | 0.50-1.61 |  | 0.92 | 0.51-1.53 |  |
| Past abdominal surgery | 0.92 | 0.79-1.08 |  | 0.95 | 0.83-1.09 |  | 0.97 | 0.85-1.11 |  |
| Surgical site infection  by 30 POD | 3.53 | 2.18-5.38 |  | 4.43 | 3.01-6.28 |  | 4.83 | 3.39-6.67 |  |

OR: odds ratio; CI: confidence interval; LSG: laparoscopic sleeve gastrectomy; LRYGB: laparoscopic Roux-en-Y gastric bypass; SVI: Social Vulnerability Index; AFib: atrial fibrillation; MI: myocardial infarction

**Supplemental Table 5.** Multivariable analyses of other predictors of major bleeding events at 30, 60, and 90 postoperative days (POD)

|  | **POD 30** | |  | **POD 60** | |  | **POD 90** | |  |
| --- | --- | --- | --- | --- | --- | --- | --- | --- | --- |
| **Parameter** | **OR** | **95% CI** |  | **OR** | **95% CI** |  | **OR** | **95% CI** |  |
| Age ≥ 60 | 1.25 | 1.02-1.51 |  | 1.24 | 1.04-1.48 |  | 1.30 | 1.10-1.52 |  |
| Male sex | 2.12 | 1.80-2.48 |  | 2.09 | 1.81-2.41 |  | 1.95 | 1.70-2.23 |  |
| LSG (vs. LRYGB) | 0.27 | 0.23-0.32 |  | 0.28 | 0.24-0.32 |  | 0.28 | 0.25-0.32 |  |
| SVI | 1.11 | 0.86-1.43 |  | 1.19 | 0.95-1.50 |  | 1.16 | 0.94-1.44 |  |
| Pre-op BMI ≥ 40 | 0.96 | 0.81-1.14 |  | 0.96 | 0.83-1.12 |  | 0.93 | 0.81-1.07 |  |
| Peri-op prophylaxis | 0.97 | 0.83-1.14 |  | 1.02 | 0.88-1.17 |  | 1.00 | 0.88-1.15 |  |
| Prior VTE | 1.98 | 1.51-2.55 |  | 1.92 | 1.50-2.42 |  | 1.75 | 1.39-2.19 |  |
| Prior anticoagulation | 1.33 | 1.04-1.69 |  | 1.24 | 0.99-1.54 |  | 1.27 | 1.03-1.56 |  |
| History of diabetes | 1.13 | 0.97-1.31 |  | 1.14 | 1.00-1.31 |  | 1.16 | 1.02-1.31 |  |
| History of AFib | 2.22 | 1.67-2.91 |  | 2.21 | 1.71-2.82 |  | 2.13 | 1.68-2.68 |  |
| History of anemia | 1.21 | 1.03-1.42 |  | 1.25 | 1.08-1.44 |  | 1.28 | 1.12-1.46 |  |
| History of MI | 1.67 | 1.07-2.46 |  | 1.81 | 1.25-2.55 |  | 2.08 | 1.50-2.81 |  |
| History of renal failure | 1.39 | 1.10-1.74 |  | 1.46 | 1.18-1.78 |  | 1.48 | 1.23-1.79 |  |
| History of dialysis | 0.83 | 0.32-1.76 |  | 1.38 | 0.74-2.02 |  | 1.63 | 0.96-2.61 |  |
| Past abdominal surgery | 1.36 | 1.16-1.59 |  | 1.41 | 1.22-1.62 |  | 1.40 | 1.23-1.60 |  |
| Surgical site infection  by 30 POD | 2.51 | 1.37-4.18 |  | 2.34 | 1.33-3.78 |  | 2.54 | 1.54-3.90 |  |

OR: odds ratio; CI: confidence interval; LSG: laparoscopic sleeve gastrectomy; LRYGB: laparoscopic Roux-en-Y gastric bypass; SVI: Social Vulnerability Index; AFib: atrial fibrillation; MI: myocardial infarction

|  | | **Venous Thromboembolism** | | | | | | |  |  | | | | **Major Bleeding Event** | | | | | |  | |
| --- | --- | --- | --- | --- | --- | --- | --- | --- | --- | --- | --- | --- | --- | --- | --- | --- | --- | --- | --- | --- | --- |
|  | **POD 30** | |  | **POD 60** | |  | **POD 90** | | | |  | **POD 30** | | |  | **POD 60** | |  | **POD 90** | |  |
| **Regimen** | **n (%)** | **p** |  | **n (%)** | **p** |  | **n (%)** | **p** | | |  | **n (%)** | **p** | |  | **n (%)** | **p** |  | **n (%)** | **p** |  |
| Apixaban 2.5mg BID |  |  |  |  |  |  |  |  | | |  |  |  | |  |  |  |  |  |  |  |
| LSG | 12 (0.23) | 1 |  | 17 (0.32) | 0.677 |  | 20 (0.38) | 0.584 | | |  | *** (<0.21) | 0.094 | |  | *** (<0.21) | 0.135 |  | *** (<0.21) | 0.067 |  |
| LRYGB | *** (<1.01) |  |  | *** (<1.01) |  |  | *** (<1.01) |  |  |  |  | *** (<1.01) |  |  |  | *** (<1.01) |  |  | *** (<1.01) |  |  |
| Rivaroxaban 10mg QD |  |  |  |  |  |  |  |  | | |  |  |  | |  |  |  |  |  |  |  |
| LSG | *** (<0.18) | 1 |  | *** (0.23) | 0.984 |  | *** (0.35) | 0.747 | | |  | *** (<0.18) | 0.908 | |  | *** (0.19) | 1 |  | *** (0.27) | 1 |  |
| LRYGB | 0 (0.00) |  |  | 0 (0.00) |  |  | 0 (0.00) |  |  |  |  | *** (0.41) |  |  |  | *** (0.41) |  |  | *** (0.41) |  |  |

**Supplemental Table 6.** Outcomes within 30, 60, and 90 postoperative days (POD) by procedure for select post-discharge chemoprophylaxis regimens

Significance based on χ² test comparing LRYGB vs. LSG within each regimen and timepoint. BID: twice daily; QD: once daily; LSG: laparoscopic sleeve gastrectomy; LRYGB: laparoscopic Roux-en-Y gastric bypass; *** Indicates suppressed count per CMS policy

**Supplemental Figure 1.** Covariate balance before and after propensity score matching


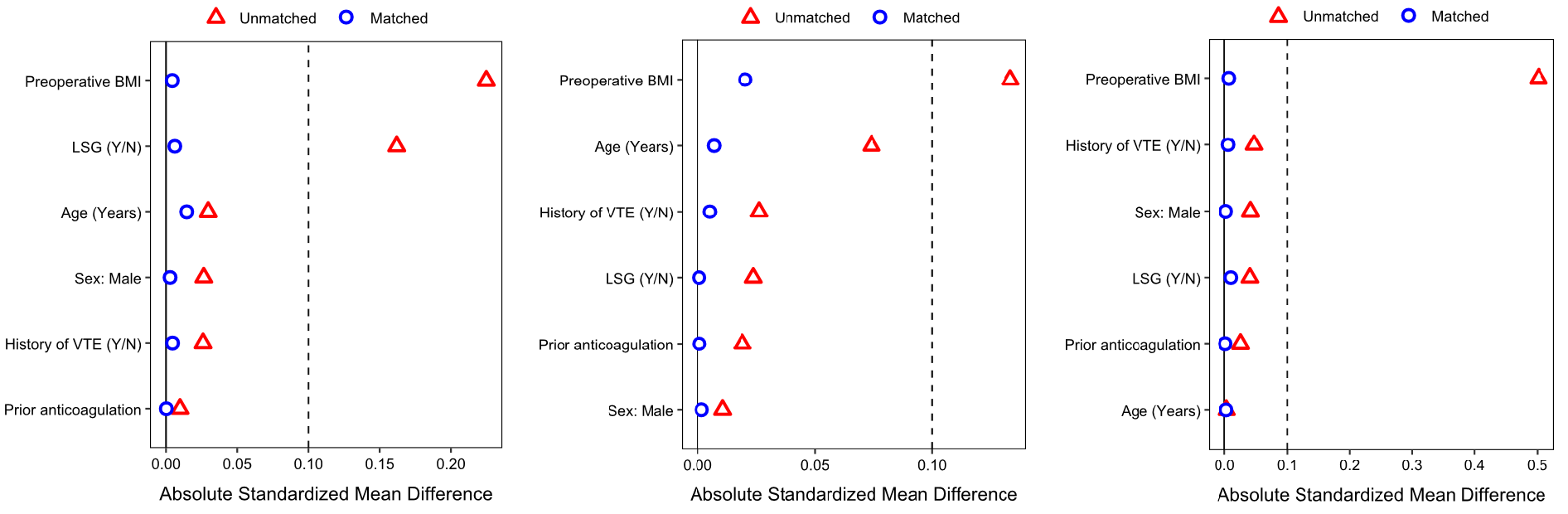


**Apixaban 2.5 mg BID**

**Enoxaparin 40 mg QD**

**Enoxaparin 40 mg BID**

BMI: Body Mass Index; LSG: laparoscopic sleeve gastrectomy; VTE: venous thromboembolism; Vertical dashed lines indicate a standardized mean difference of 0.1, which was used as the pre-specified threshold for acceptable covariate balance.
